# Among the Colleges

**Published:** 1901-04

**Authors:** 


### AMONG THE COLLEGES.

UNIVERSITY OF PENNSYLVANIA VETERINARY DEPARTMENT. The Veterinary Medical Association of this University gave an informal smoker at the reading room of the department, Friday evening, March 1, 1901. The several classes to the number of fifty were in attendance, and amid the soothing influences of the corncob pipes, decorated with ribbons carrying the University colors, and light refreshments, a very enjoyable time was experienced. Dr. Adams as a speech-maker was fair, but as a story-teller was unsurpassed, and entertained the boys until midnight. Dr. C. J. Marshall, as an invited guest, thought they did not have these smokers often enough. Editor Hoskins, of the JOURNAL, addressed the boys on their future work, but enjoyed Adams' stories until the last one was told. Drs. Pearson and Cornman were also among the invited guests.

ONTARIO VETERINARY COLLEGE. This college has had quite a successful session which has not yet closed. We fully recognize that veterinary education does not merely consist in stuffing the mind with facts, but in cultivating the powers of reason, observation, and reflection as well as memory. All the various branches of knowledge appertaining to veterinary science was dealt with, and the teachings, both theoretical and practical, have kept pace with the times.

The veterinary association in connection with the college, consisting of the students and staff, has held its regular meetings, over one hundred and thirty papers contributed by the students having been already read, and there are several more to follow. Many of these have been of much interest, and have been freely discussed. They embrace a great variety of subjects, several that have come under the personal observation of the contributors. These papers and the discussions on them that follow are viewed with much interest by the students, and cannot fail to be of inestimable benefit.

VETERINARY DEPARTMENT, IOWA AGRICULTURAL COLLEGE. Since September, 1900, the Veterinary Department of the Iowa State College of Agriculture and the Mechanic Arts has undergone a complete reorganization and has been infused with new life. Dr. M. Stalker has been retired from the deanship and is now no longer a factor in the affairs of the department. With the accession of Dr. John J. Repp to the teaching force in January, 1899, as Professor of Pathology and Therapeutics, a movement was begun looking toward the amelioration of the condition of the school. In September, 1900, the teaching force was increased by the election of Dr. John H. McNeall as Professor of Anatomy and Surgery, and Dr. Louis A. Klein as Professor of Theory and Practice of Medicine and Sanitary Science. The school now has a larger and stronger teaching force than at any previous time in its entire history. It is able to avail itself of the excellent facilities offered by the departments of chemistry, botany, zoölogy, and agriculture of the college with which it is connected. A course in pharmacy was introduced into the curriculum for the first time in the life of the school last September. The hospital work has been systematized in such a way as to contribute the utmost to the instruction of the student. The clinics and hospital practice are now larger and better than ever. The curriculum has been materially reconstructed and very greatly strengthened. The teaching of the various branches of the course has been harmonized, and the standard of education has been measurably advanced. The course of study is not excelled anywhere in America.

The school is happy in the enjoyment of the confidence and support of the profession of Iowa and surrounding States.

Now that the veterinary department has begun to grow, it is to be earnestly hoped that those charged with its management will put plenty of money and energy into it, and rapidly push it forward to the front rank of veterinary schools in America. This school is fortunate in its location in the centre of a group of the best agricultural and stock-raising States in the Union.

Veterinary Department of McGill University. Dr. Y. Kato, who graduated a year ago, after a few months visiting in Japan, has returned for a post-graduate course, and has been working in the pathological laboratories and with Dr. Higgins.

Dr. Higgins, assistant pathologist on the cattle quarantine staff, returned at the beginning of the year from British Columbia, where he had been temporarily transferred to the medical quarantine to prepare antitoxines for use at that station. He has been appointed assistant to the professor of pathology in the faculty of comparative medicine and veterinary science.

Dr. Angus Tracy, since returning six weeks ago, has been hard at work in the pathological laboratories at McGill University.

Dr. B. A. Sugden was recently appointed lecturer on materia medica in the faculty. At the close of the session he was entertained by the students at dinner, and was presented with a handsome testimonial.

Graduates in the Boer war.—The following took part in the war and on transportation of horses and mules, viz.:

Dr. T. Wroughton, as veterinary surgeon, to first royal Canadian mounted infantry; Dr. Angus Tracy, to first royal Canadian mounted infantry; Dr. G. T. Stevenson, Strathcomas horse; Dr. W. B. Wallis, V.S., to imperial yeomanry; Dr. I. P. Spanton, V.S., to imperial yeomanry; Dr. G. M. Blanchard, as lieutenant Canadian infantry, died of his wounds in South Africa.

In the transport service:

Dr. Joseph Plaskett made three very successful trips; Dr. John M. Parker is at present in South Africa engaged in transport service; Dr. William Patterson made one trip on transport service.

The following have been appointed to the permanent staff of Canadian cattle quarantine service for the purpose of testing cattle for export to the United States: Drs. A. E. Moore, C. Higgins, V. T. Daubigny, Montreal; Dr. J. A. Couture, Quebec; Dr. W. Jakeman, Halifax; J. H. Frick, V.S., St. John, N. B.; W. H. Pethick, V.S., Prince Edward Island; G. W. Higginson, V.S., Rockland, Ontario; W. Stubbs, V.S., Toronto, Ontario; Charles Little, V.S., Winnipeg, Man.; John Hargrave, D.V.S., Northwest Territory; J. B. Hart, D.V.S., Vancouver, B. C.

Testing for export to the United States in now restricted to the above-named veterinarians.

The dean of the Faculty of Comparative Medicine entertained the entire staff of the faculty, Principal Peterson, and the deans of the other faculties to a very enjoyable dinner at his city residence on the evening of the convention for conferring degrees.

The tuberculosis conference to be held in London, England, on July 22d promises to be a very important meeting. It is expected that the king will as president open the conference in person. Representative men will attend from all the European countries, including such well-known men as Bangs, Nocard, Koch, Roux, etc.

The United States and Canada will be represented, the latter by the dean of the Faculty of Comparative Medicine, who is vice-president of the animal section, and will open the discussion on "Legislation for the Control of Tuberculosis." Prof. Adami, as vice-president of the pathological section, and several other medical professors from Canada, will also attend.

Chicago Veterinary College. On March 5th the annual banquet of the faculty and students of the Chicago Veterinary College was given by the trustees at the Sherman House. Ninety-five students and the faculty of twelve partook of the good things set before them. Dr. A. H. Baker acted as toast-master, and speeches were made by Dr. E. M. Bronson, representing the senior class; Mr. L. S. Robertson, of the junior class, and Mr. E. L. Lewis, of the freshman. Dr. A. S. Alexander spoke for the faculty. Several speeches were made by other members of the faculty and students, interspersed with vocal and instrumental selections by members of the class, and a whistling solo with piano accompaniment was given by Prof. G. M. Cushing. Being a family affair, undue restraint was noticeably absent, and each one enjoyed himself heartily.

